# Recent advances in metal catalyst- and oxidant-free electrochemical C-H bond functionalization of nitrogen-containing heterocycles

**DOI:** 10.3389/fchem.2022.967501

**Published:** 2022-08-19

**Authors:** Ya-Nan Li, Bin Wang, Ye-Kai Huang, Jin-Song Hu, Jia-Nan Sun

**Affiliations:** ^1^ School of Chemical Engineering, Anhui University of Science and Technology, Huainan, China; ^2^ School of Biomedical Engineering, Research and Engineering Center of Biomedical Materials, Anhui Medical University, Hefei, China

**Keywords:** electrochemical, C-H bond functionalization, nitrogen-containing heterocycles, oxidant-free, metal catalyst-free

## Abstract

The C-H functionalization of nitrogen-containing heterocycles has emerged as a powerful strategy for the construction of carbon-carbon (C-C) and carbon-heteroatom (C-X) bonds. In order to achieve efficient and selective C-H functionalization, electrochemical synthesis has attracted increasing attention. Because electrochemical anodic oxidation is ideal for replacing chemical reagents in C-H functionalization reactions. This mini-review summarizes the current knowledge and recent advances since 2017 in the synthetic utility of electrochemical transformations for the C-H functionalization of nitrogen-containing heterocycles.

## Introduction

*N-*heterocyclic structures are widely found in biologically active natural products, drug molecules, agrochemicals, and functional materials (Goel et al., 2016). For example, approximately more than 60% of the FDA-approved small molecules bear at least one nitrogen heterocycle (Vitaku et al., 2014). Therefore, approaches to functionalize existing C-H bonds of nitrogen heterocycles has continuously attracted attention of organic chemists (Sharma et al., 2013). Most of the classical C-H bond functionalization is catalyzed by metal catalysts such as Rh, Pd, Ni, Mn, Co, Ir, Cu, Ag with concomitant ligand (Davies et al., 2017). Although these methods have achieved good results, there are many limitations. On the one hand, the conventional methods usually required the use of expensive catalysts or toxic oxidants. On the other hand, most of these approaches involved harsh conditions and the generation of large amounts of waste. Besides, the direct C-H functionalization reactions with heterocyclic structures also tend to be challenging due to the strongly coordinating nature of the nitrogen atom, which can poison the metal catalysts or activate undesired positions. In this regard, more economical and green synthetic approaches for the C-H functionalization of nitrogen heterocycles are continuously and highly required.

Electrochemistry has been an efficient strategy for organic synthesis (Jiang et al., 2018; Li et al., 2021). Compared with the conventional methods of the C-H functionalization of nitrogen heterocycles, the electrochemistry method uses electron as an inexpensive, renewable, and environmentally friendly reagent to replace the stoichiometric amounts of chemical regent in the C-H functionalization reactions. Besides, some typical oxidative transformations could be achieved since the reaction reactivity could be tuned by changing the electrode potential. Undoubtedly, the electrochemical C-H bond functionalization of nitrogen heterocycles was considered for a long time as a sustainable technique for synthetic chemistry.

**FIGURE 1:**
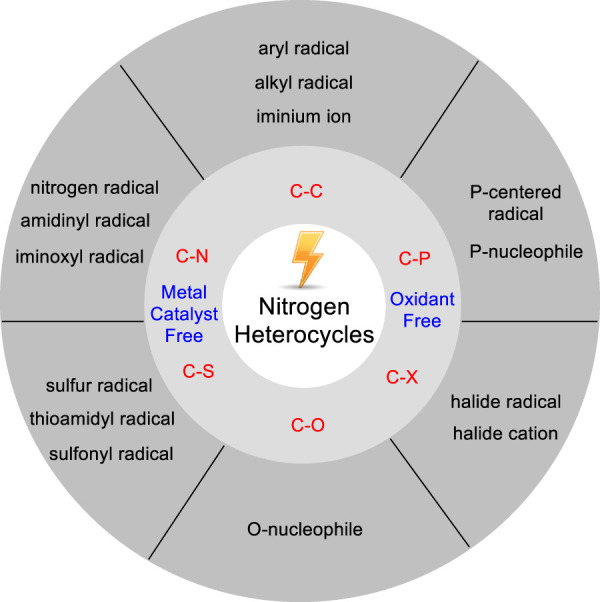
Electrochemical C-H Bond Functionalization of Nitrogen Heterocycles.

Some reviews on the electrochemical functionalization of С-Н bonds in heterocycles have been reported so far. Chupakhin and co-workers (Chupakhin et al., 2020) wrote a review on application of electrochemical oxidative methods in the C (*sp* ^*2*^)-H functionalization of heterocyclic compounds in 2019. Meanwhile, Kakiuchi (Kakiuchi and Kochi., 2020) summarized the progress of the catalytic oxidative C-H functionalization in efficient combination of transition-metal catalyst and electrochemical oxidation until 2019. Recently, Budnikova (Budnikova, 2021) outlined a comprehensive advance in regard to electrochemical insight into mechanism and metallocyclic intermediates of C-H functionalization. In this mini-review, we will summarize recent advances since 2017 in metal catalyst-free electrochemical C-H bond functionalization of nitrogen-containing heterocycles. This mini-review is classified into six sections according to the different C-H functionalization of nitrogen-containing heterocycles, and only selected examples are described schematically (Figure 1).

### C-C bond formation

The catalytic formation of C-C bonds based on C-H functionalization is one of the most efficient atom-step economic reactions for the synthesis of complex molecules. Various methods have been developed to replace C-H bonds with transition metal to form C-C bonds (Ohno et al., 2021). Most of these reactions use high-valent transition metal catalysts and stoichiometric amounts of chemical oxidants. In this section, we focus on metal catalyst-free electrochemical C-H bond functionalization of nitrogen heterocycles to form C-C bonds.

**SCHEME 1:**
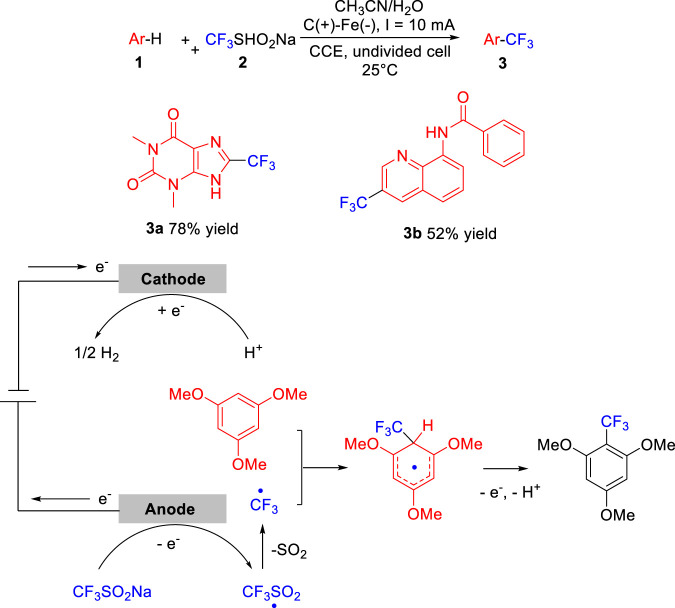
Electrochemical C-H Trifluoromethylation of Arenes.

In 2019, Lei group (Deng et al., 2019) reported an efficient and environmentally benign metal catalyst-free electrochemical oxidative radical C-H trifluoromethylation of arenes **1** by employing Langlois reagent **2** as the CF_3_ source. The trifluoromethylation reaction was conducted in an undivided cell equipped with a carbon rod anode and an iron plate cathode at a constant current of 10 mA using LiClO_4_/*n*-Bu_4_NBF_4_ as the electrolyte at 25°C. As illustrated in Scheme 1, theophylline **1a** and *N*-(quinolin-8-yl)-benzamide **1b** were selectively functionalized to produce the corresponding trifluoromethylation products **3a** and **3b** in 78 and 52% yield, respectively. Control experiments suggested that the electrochemical trifluoromethylation of arenes critically involved the generation of **CF**_**3**_ radical by anodized at the anode in a desulfurative manner. The scope of the substrates was investigated, it could be seen that this reaction complies with the substituent positioning effect. **CF**_**3**_ radical generally attacked the *ortho-* and *para-*position of electron-donating groups of aromatic rings such as methyl group and methoxy group.

**SCHEME 2:**
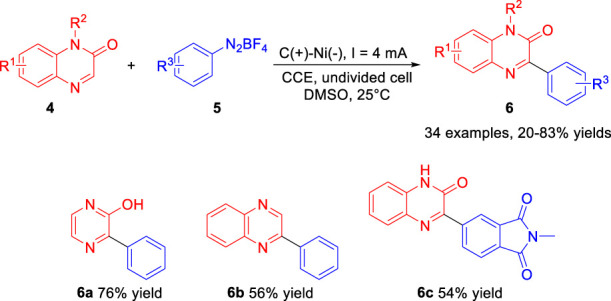
Electrochemical Arylation Reaction of C (*sp* ^*2*^)-H with Aryldiazonium Salts.

Also in 2019, Zeng and co-workers (Jiang et al., 2019) developed an efficient electrochemical approach to the Minisci-type arylation reaction of C (*sp* ^*2*^)-H with aryldiazonium salts **5**. The paired electrosynthesis proceeds in a simple undivided cell equipped with a graphite anode and a Ni plate cathode at a constant current of 4 mA lacking supporting electrolyte at 25°C. As illustrated in Scheme 2, a variety of arylated products **6** including the electron-donating and electron-withdrawing groups, were afforded in 20–83% yields. Notably, when pyrazin-2-ol and electron-deficient heterocycle quinoxaline were used as the substrate, the corresponding products **6a** and **6b** were obtained in 76% yield and 56% yield, respectively. In addition, the heterocyclic aromatic diazonium salt could also be smoothly transformed into the desired products **6c** in 54% yield. Based on controlled experiments, the authors proposed that the arylation reaction underwent the process of the aryl radical, which was gained from the phenyl diazo radicals losing a molecule of nitrogen.

**SCHEME 3:**
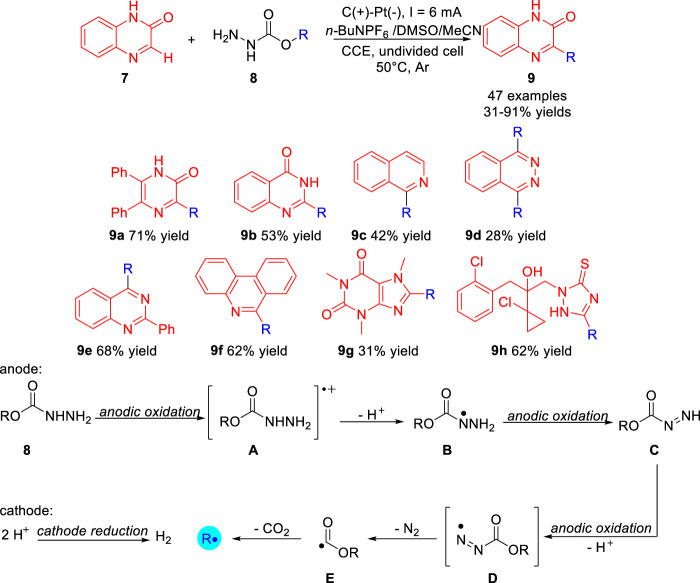
Electrochemical Deoxygenative Functionalization of Heteroarenes.

In 2020, Wang group (Gao P S et al., 2020) reported the carbazate as a new type of electrochemically activated alkylating agent **8** for the direct functionalization of heteroarenes **7** under electrochemical conditions. A variety of primary, secondary and tertiary alkyl-decorated heterocycles **9** were obtained with 31–91% yields in a simple undivided cell at a constant current of 4 mA with carbon/platinum electrode set-ups, using *n*-Bu_4_NPF_6_ as an electrolyte at 50°C under the protection of argon. As presented in Scheme 3, the heterocycles including pyrazinone, quinazolinone, isoquinoline, phthalazine, quinazoline, and phenanthridine could also be carried out smoothly to give the desired corresponding alkylated products. Notably, the bioactive compounds caffeine and prothioconazole were also compatible with the reaction system, the corresponding products **9g** and **9h** were obtained in 31% yield and 62% yield, respectively. Control experiments suggested that the electrochemical deoxyalkylation reaction involved the continuous oxidation of **8** to generate the alkyl radical **R** at the anode. The first stage is the continuous anodization and deprotonation of carbamate **8** to form diazenecarboxylate **C**. Further anodic oxidation of **C** cleaves diazene to generate acyl radical **E** while releasing molecular nitrogen. The second step is decarboxylation of acyl radical **E** to generate alkyl radical **R** at the anode.

**SCHEME 4:**
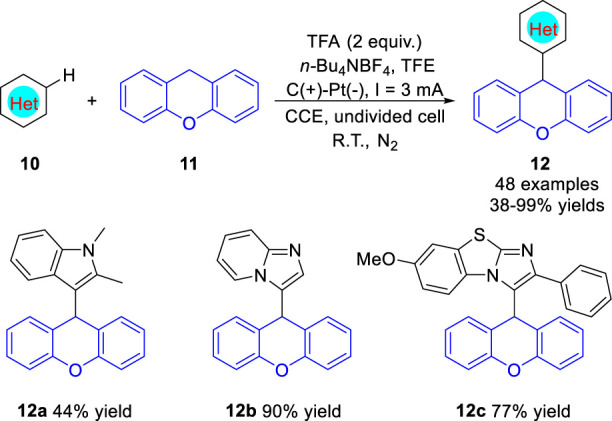
Electrooxidation-Induced C-H Alkylation of Electron-rich Arenes.

Lei group (Liang et al., 2022) reported an efficient electrooxidation-induced radical C (*sp* ^*3*^)-H arylation of xanthenes **11** with electron-rich arenes **10** under metal catalyst- and oxidant-free conditions in 2022. The electrosynthesis proceeds in a simple undivided cell equipped with a graphite anode and a Pt plate cathode at a constant current of 3 mA, using *n*-Bu_4_NBF_4_ as an electrolyte at room temperature under the protection of nitrogen. Notably, the nitrogen-containing heterocyclic compounds, such as *N*-methylindole, imidazo [1,2-*a*]pyridine and benzo [*d*]-imidazo [2,1-*b*] thiazole, were successfully employed as the reaction substrate under standard conditions, and the corresponding products **12a-12c** were obtained in moderate to good yields, respectively (Scheme 4). The reaction involves cross-coupling of free radicals to form C (*sp* ^*3*^)-C (*sp* ^*2*^) bonds, which oxidized concurrently via SET process at the anode. When *N*-alkyl substituted anilines were employed as the substrates, xanthene was substituted at the *para*-position of the amino group. The proposed mechanism indicated that *N*-methylaniline radical which electron located in *para*-position of the amino group was generated.

**SCHEME 5:**
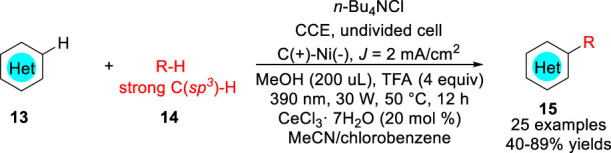
Electrophotocatalytic C-H Alkylation of *N*-heteroarenes.

Recently, Xu, Zeng and co-workers (Tan et al., 2022) reported a new electrophotocatalytic C-H functionalization of *N*-Heteroarenes **13** with unactivated alkanes **14** under external oxidant-free conditions with H_2_ evolution (Scheme 5). A variety of alkylated *N*-heteroarenes **15** including isoquinoline, quinoline, quinoxalinone, pyridine, phenanthridine, and benzothiazole were obtained with 40–89% yields in an undivided cell equipped with a carbon anode and a Ni foam cathode with inexpensive CeCl_3_ 7H_2_O as the catalyst under photoirradiation (390 nm, 30 W). Control experiments suggested that the electrophotocatalytic alkylation reaction involved the oxidation of **14** to generate the alkyl radicals **R** from strong C (*sp* ^*3*^)-H bonds at the anode.

**SCHEME 6:**
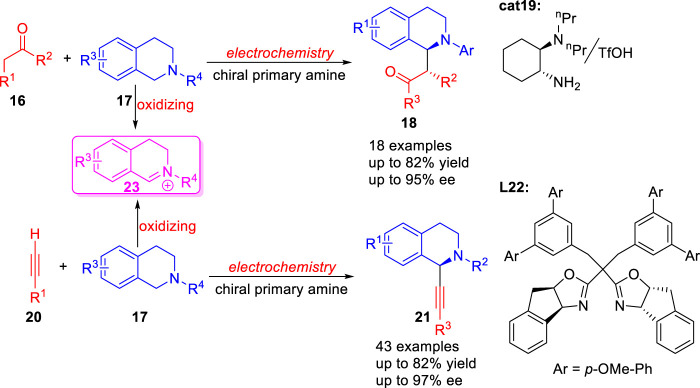
Electrochemically Enantioselective Alkylation of Tertiary Cyclic Amines.

In 2017, Luo group (Fu et al., 2017) reported a green synthetic method for the catalytic asymmetric coupling of tertiary amines **17** with simple ketones **16** via the combination of electrochemical oxidation and chiral primary amine catalysis **19** under mild conditions (Scheme 6). Subsequently, a novel strategy for asymmetric Shono-type oxidative cross-coupling reaction of tertiary amines **17** and terminal alkynes **20** using **L22** as ligand was reported by Mei and co-workers (Gao Y Y et al., 2020) under electrochemical conditions (Scheme 6). Both reactions involved the oxidation of a tetrahydroisoquinoline **17** to an electrophilic iminium ion species **23** at the anode.

### C-N bond formation

The direct electrochemical functionalization of C-H bonds with Nitrogen Heterocycles can serve as one of the most powerful tools to directly construct C-N bonds in synthesis due to requirements of “green chemistry”, and has also provided an innovative alternative to the metal-catalyzed C-H functionalization (Zhang and Lei, 2019; Meng et al., 2021a).

**SCHEME 7:**
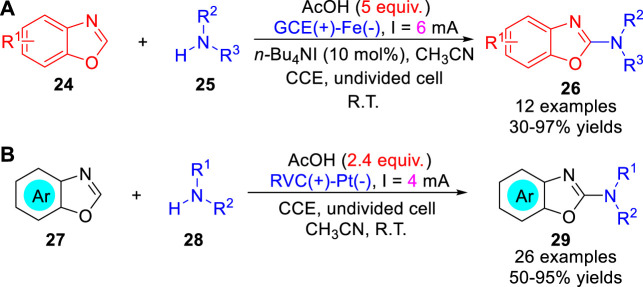
Electrochemically Oxidative C-H Amination of Benzoxazoles.

In 2014, Zeng, Little and co-workers (Gao et al., 2014) reported an electrochemically oxidative amination of benzoxazoles **24** with amines **25** using tetraalkylammonium halide redox catalyst avoiding large amounts of electrolyte. A variety of substrates including morpholine, cyclic and acyclic secondary amines were successfully used in the amination of benzoxazoles to afford the corresponding 2-aminobenzoxazoles **26** in 30–97% yields under constant current conditions in a simple undivided cell (Scheme 7a). However, primary amines and phthalimide failed to react under the optimized reaction conditions. The reaction involved the electrochemical oxidation of halide to forms **I** ^**+**^, which reacted with the intermediate benzoxazoline. Subsequently, the same reaction was also realized through direct electrosynthesis by Ackermann et al. (Qiu et al., 2018). The electrosynthesis proceeds in a simple undivided cell equipped with a reticulated vitreous carbon (RVC) anode and a platinum plate cathode at a constant current of 4 mA under catalyst-, electrolyte- and chemical oxidant-free conditions. In addition to morpholine, cyclic and acyclic secondary amines were successfully used in this reaction, anilines were also compatible with the reaction (Scheme 7b). In contrast, the electrochemical azole C-H amination reaction was proposed to initiate via anodic oxidation of amine **28**.

**SCHEME 8:**
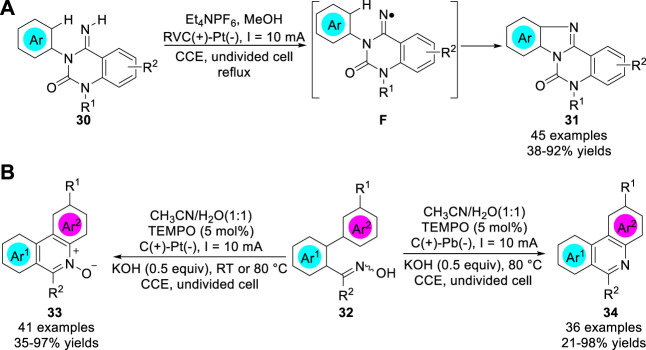
Intramolecular Electrochemical Amination to Prepare Nitrogen Heterocycles.

In 2017, Xu group (Zhao et al., 2017) reported a metal catalyst-free electrochemical approach to access amidinyl radical intermediates, which underwent cyclizations with (hetero) arenes **30**, followed by rearomatization, to generate functionalized tetracyclic benzimidazoles and pyridoimidazole **31** in 38–92% yields (Scheme 8a). The cyclization of reactive nitrogen-centered radicals (NCRs) reaction was conducted in an undivided cell equipped with a RVC anode and a Pt plate cathode at a constant current of 10 mA using Et_4_NPF_6_ as the electrolyte. Controlled experiments showed that this reaction involved a process of an amidinyl radical intermediate **F**. Subsequently, the same group (Zhao et al., 2018) further developed the electrolysis of polycyclic *N*-heteroaromatic compounds **34** and their corresponding N-oxides **33** through electrochemical C-H functionalization of biaryl ketoximes **32** by employing different cathode materials (Scheme 8b). Control experiments suggested that an iminoxyl radical was involved in the reaction pathway. Interestingly, the study of the scope of the substrates indicated that the *para*-position R^1^ were *para*-directing groups, such as methoxy group or halogen groups, the yields of corresponding products were significantly higher than *meta*-directing ester group.

**SCHEME 9:**
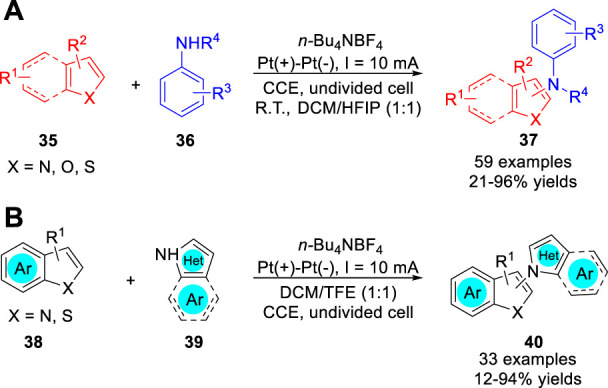
Electrochemically Oxidative Amination of Heteroarenes.

In 2021, Ruan, Feng and co-workers (Peng et al., 2021) reported an effective C-H amination of heteroarenes **35** with aniline derivatives **36** *via* an electro-oxidative cross-coupling process. A variety of substrates including indole, thiophene, benzothiophene, benzofuran and pyrrole were successfully used in the amination reaction to afford the corresponding aminated five-membered heteroarenes **37** in 21–96% yields under constant current conditions in a simple undivided cell (Scheme 9a). Control experiments suggested that a radical-radical cross-coupling process between the *N*-centered radical and cationic indole radical may be involved for this amination reaction. Very recently, the same group (Zhou et al., 2022) further developed an electrochemical oxidative coupling of azoles **38** with 2- and 3-Haloindoles/Thiophenes **39** under metal catalyst- and oxidant-free conditions. A series of C-2/C-3 aminated halo-indoles/thiophenes **40** were obtained in a simple undivided cell equipped with two platinum electrodes at a constant current of 10 mA, using *n-*Bu_4_NBF_4_ as an electrolyte (Scheme 9b). Based on controlled experiments, the authors proposed that the amination reaction involves the oxidative C-H/N-H cross-coupling process between the pyrazole radical and cationic indole radical.

**SCHEME 10:**
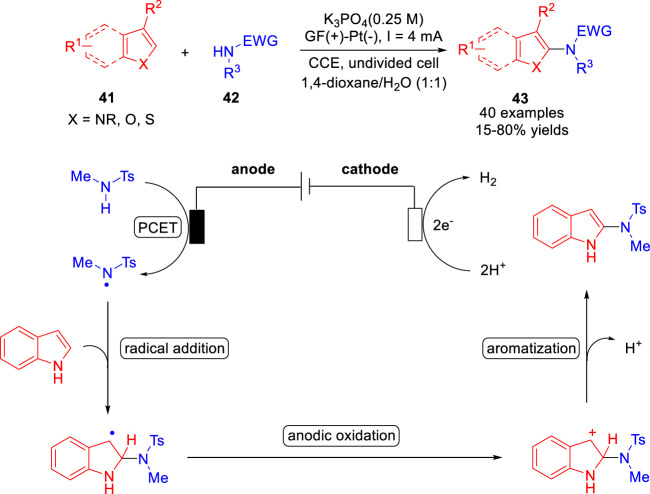
Electrochemically Oxidative Amination of Heteroarenes.

The Zhang, Ackermann and co-workers (Zhang et al., 2021) reported catalyst and chemical-oxidant-free electrochemical C-H amidation of heteroarenes **41** with *N*-alkyl sulfonamides **42** in aqueous medium. A variety of heteroarenes, including indoles, pyrroles, benzofurans and benzothiophenes, were successfully used in the amination reaction to form C-N bonds in an undivided cell equipped with a GF anode and a Pt plate cathode at a constant current of 4 mA using K_3_PO_4_ as the base (Scheme 10). Besides, when the substrate heteroarene was changed to naphthalene, the desired product was also obtained in 15% yields. Detailed experiments and cyclic voltammetry studies suggested that a nitrogen radical was involved in the reaction pathway *via* an anodic oxidation.

**SCHEME 11:**
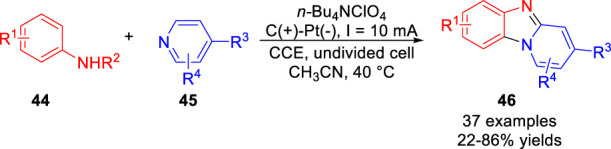
Electrochemical [3 + 2] heteroannulation of Anilines with Pyridines.

In 2021, the metal catalyst-free electrochemical [3 + 2] heteroannulation of anilines **44** with pyridines **45** was realized via dual C-H radical aminations by Li *et al* (Luo et al., 2021). A variety of functionally diverse benzo [4,5] imidazo [1,2-*a*]pyridines **46** were obtained with moderate to good yields in an undivided cell equipped with a graphite rod anode and a Pt plate cathode at a constant current of 10 mA using *n*-Bu_4_NClO_4_ as the electrolyte (Scheme 11). The electron-withdrawing groups at the 4-position of the pyridine ring are crucial to initiate the heteroannulation reaction, and control experiments suggested that a radical coupling process between the nitrogen-centered radical and the pyridine radical might be involved in this electrochemical transformation.

### C-O bond formation

**SCHEME 12:**
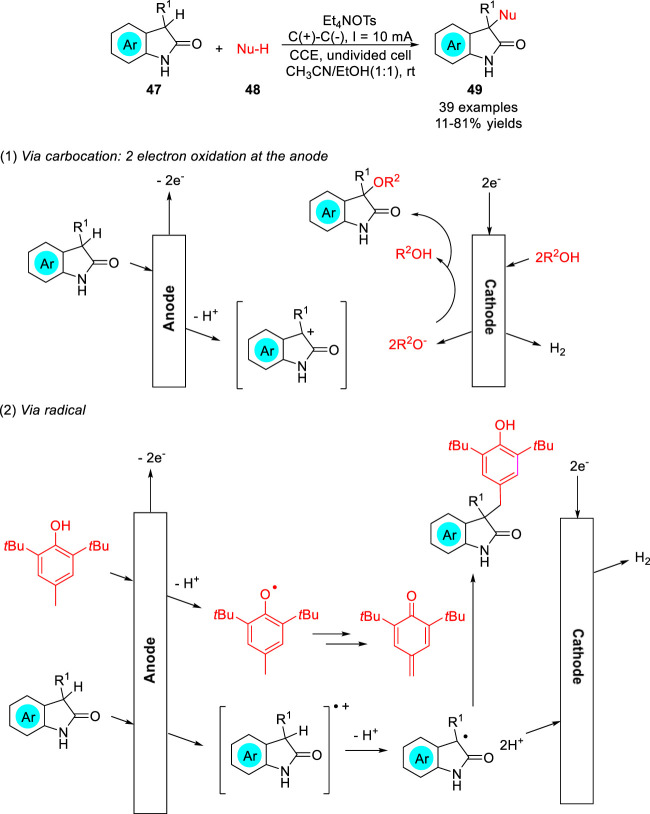
Electrochemical C-H Functionalization of Oxindoles.

The electrochemical C-H bond functionalization of nitrogen heterocycles using O-nucleophile to construct C-O bonds was quite rarely. The direct electrochemical umpolung C-H functionalization of oxindoles **47** was realized in mixed ethanol and acetonitrile solvent by the Maulide group (Pastor et al., 2022) to construct C-O bonds under mild environmentally benign conditions, and this synthetic method could also be extended to C-C or even C-N bond formation. A variety of unsymmetrical 3,3-disubstituted oxindoles **49** were obtained with moderate to good yields in an undivided cell equipped with two graphite electrodes at a constant current of 10 mA using Et_4_NOTs as the electrolyte (Scheme 12). Depending on the control experiment, the reaction may involve the two-electron oxidized carbocation or the single-electron oxidized radical cation of the oxindole at the anode.

### C-P bond formation

Phosphorus containing compounds are of great importance due to their broad application in synthetic chemistry, agricultural chemicals, natural products, and material science (Demmer et al., 2011). Continued efforts have been devoted to the development of their synthesis, among which the direct electrochemical oxidative C-H phosphonylation is recognized as an efficient approach for the construction of organophosphorus compounds compared to conventional methods to avoid using metal catalysts, stoichiometric oxidants and pre-functionalized starting materials.

**SCHEME 13:**
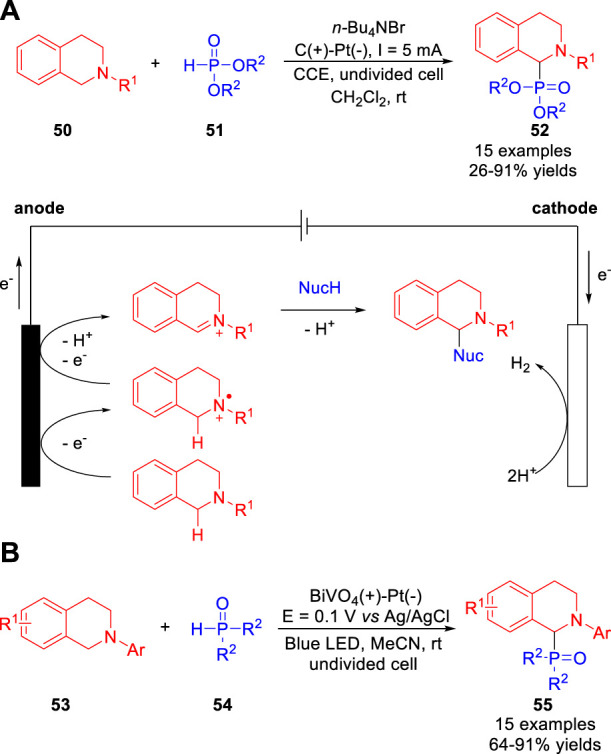
Electrochemical Oxidative C-H Phosphonylation of *N*-aryltetrahydroisoquinolines.

In 2019, the Cui, Xiang and co-workers (Xie et al., 2019) reported a metal catalyst- and reagent-free electrochemical cross-dehydrogenative coupling reaction of *N*-aryl-tetrahydroisoquinolines **50** with phosphites **51**, and the aminophosphonates **52** were obtained with moderate to high yields in an undivided cell equipped with a graphite rod anode and a Pt plate cathode at a constant current of 5 mA using *n*-Bu_4_NBr as the electrolyte (Scheme 13a). Besides, the indole was also compatible with this reaction system. This reaction involved the oxidation of a *N*-aryltetrahydroisoquinoline **50** to an electrophilic iminium ion species captured by the reactive nucleophile. Subsequently, the same reaction was developed through an efficient photoelectrochemistry method by Wu et al. (Wang et al., 2019). The photoelectrosynthesis proceeds in a photoelectrochemical cell equipped with a BiVO4 as the working electrode, a Pt plate as the counter electrode and Ag/AgCl as the reference electrode at a constant potential of +0.1 V vs. Ag/AgCl using *N*-hydroxyphthalimide (NHPI) as the light mediator (Scheme 13b). As compared with an electrochemical cell, nearly 90% external bias input was saved to produce the desired aminophosphonates **55** in good to excellent yields.

**SCHEME 14:**
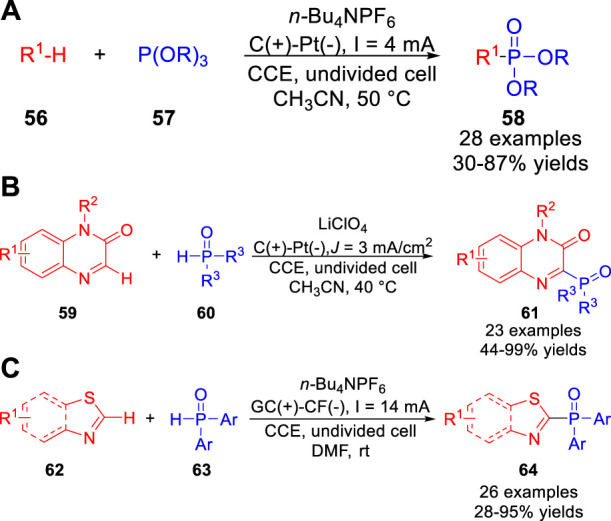
Photoelectrochemical Oxidative C-H Phosphonylation of N-aryltetrahydroisoquinolines.

The electrochemical phosphonylation was realized by the Lei group (Yuan et al., 2019a) under exogenous oxidant-free and metal catalyst-free electrochemical oxidation conditions. A variety of heteroarenes **56**, including imidazo [1,2-*a*] pyridine, benzo [d]imidazo [2,1-*b*]thiazole, benzofuran, *N*-methylindole, *p*-xylene, xanthene, *N*-methyl-9,10-dihydroacridine, *N,N*-dimethylaniline and *N*-phenyl tetrahydroisoquinoline were successfully used in the phosphonylation reaction to form C-P bonds in an undivided cell equipped with a carbon rod anode and a platinum plate cathode at a constant current of 4 mA using *n*-Bu_4_NPF_6_ as the electrolyte (Scheme 14a). Control experiments suggested that imidazo [1,2-*a*] pyridine or xanthene was oxidized to the radical cation intermediate or xanthene carbocation intermediate, which was captured by P(OR)_3_ **57** in this phosphonylation reaction, respectively. Immediately, the Zeng group (Li et al., 2019) reported a metal catalyst- and reagent-free electrochemical approach to produce 3-phosphonated quinoxalin-2(*1H*)-ones **61** with good to excellent yields. The phosphonylation reaction was conducted in an undivided cell equipped with a graphite anode and a platinum net cathode at a constant current of 3 mA cm^−2^ using LiClO_4_ as the electrolyte at 40°C (Scheme 14b). Furthermore, this protocol could also be applied to C (*sp* ^*3*^)-H phosphonation reaction of xanthenes under the standard conditions. Control experiments disclosed that this reaction may undergo the addition of a phosphorus-centered radical to the carbon-nitrogen double bond of the protonated quinoxaline-2(*1H*)-one. Recently, the Zhu, Wu and co-workers (Zhu et al., 2022) reported a direct electrochemical oxidative C-H phosphonylation of thiazole derivatives **62**. And a diverse range of phosphorus products **64** were obtained with moderate to high yields in an undivided cell equipped with a glassy carbon anode and a copper foam cathode at a constant current of 14 mA using *n*-Bu_4_NPF_6_ as the electrolyte (Scheme 14c). However, the dialkylphosphine oxides as well as diethyl phosphonate were not compatible in the current electrochemical phosphonylation system. Based on controlled experiments, the authors proposed that this reaction involved a P-centered radical pathway.

### C-S bonds formation

**SCHEME 15:**
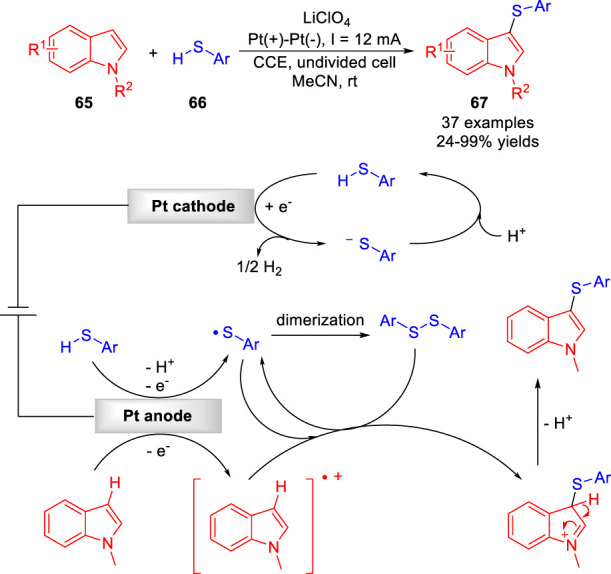
Electrochemical Oxidative C-H Phosphonylation of Heteroarenes.

The electrochemical direct C-H functionalization of nitrogen-containing heterocycles to construct C-S bonds has recently been greatly developed. In 2017, the Lei group (Wang et al., 2017) developed an environmentally friendly electrocatalytic protocol for dehydrogenative C-H/S-H cross-coupling reaction of electron-rich arenes **65** with aryl/heteroaryl thiols **66** to form C-S bond under catalyst- and oxidant-free conditions. The products of C-H thiolation **67** were obtained with moderate to high yields in an undivided cell equipped with two platinum electrodes at a constant current of 12 mA using LiClO_4_ as the electrolyte (Scheme 15). A variety of electron-rich arenes, including indoles, benzene, aniline, phenol, pyrrole, thiophene, thereby underwent this C (*sp* ^*2*^)-H thiolation. A preliminary mechanistic study suggested that the *N*-methylindole could be oxidized to generate a radical-cation intermediate, which underwent direct coupling with the sulfur radical produced by anodizing at the anode, simultaneously.

**SCHEME 16:**
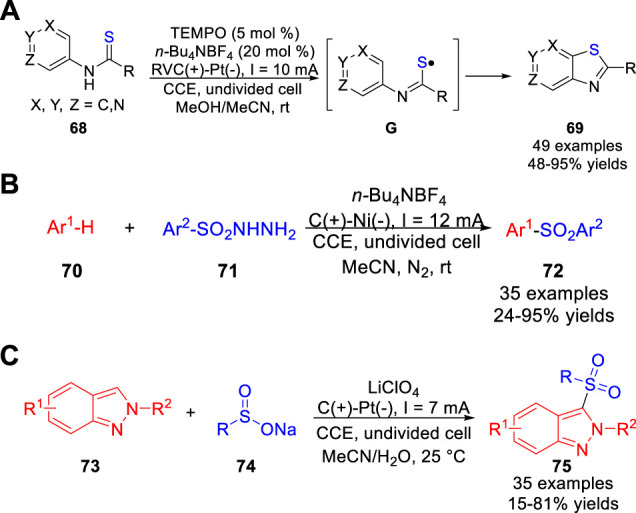
Electrochemical Oxidative C-H Phosphonylation of Quinoxalin-2(1H)-ones.

At the same time, the Song, Xu and co-workers (Qian et al., 2017) reported a TEMPO-catalyzed electrochemical C-H thiolation of *N*-(hetero) arylthioamides **68** under the metal catalyst- and reagent-free conditions. And a host of thiazole derivatives **69** were obtained with moderate to excellent yields in an undivided cell equipped with a reticulated vitreous carbon anode and a Pt plate cathode at a constant current of 10 mA using *n*-Bu_4_NBF_4_ as the electrolyte (Scheme 16a). Pleasingly, the thiazolopyridines could be obtained in good yields by employing aminopyridine-derived thioamides. It was worth mentioning that the 3-aminopyridine-based substrates could react regioselectively at the α-position of the pyridyl ring to form thiazolo [5,4,*b*] pyridines. The authors proposed that the thioamide substrate was oxidized by electrochemically generated TEMPO^+^ *via* an inner-sphere electron transfer to form an active thioamidyl radical intermediate **G**, which underwent homolytic aromatic substitution to construct C-S bond. In 2018, Lei et al. also disclosed an efficient and environmentally benign electrochemical oxidative radical C-H sulfonylation of arenes/heteroarenes **70** with arylsulfonylhydrazide **71** under mild catalyst- and exogenous oxidant-free reaction conditions (Yuan et al., 2018). And a variety of C-H sulfonylated products **72** were obtained with moderate to high yields in an undivided cell equipped with a carbon anode and a Ni cathode at a constant current of 12 mA using *n*-Bu_4_NBF_4_ as the electrolyte (Scheme 16b). Especially, the electron-rich pyrrole and imidazopyridine derivatives were also compatible with the reaction system and the corresponding products could be obtained with 36 and 62% isolated yields, respectively. Control experiments suggested that this reaction involved a sulfonyl radical pathway. In 2020, Oh and co-workers (Kim et al., 2020) studied the direct C-3 sulfonylation of *2H*-Indazoles **73** under electrochemical conditions using sodium sulfinates **74** as the sulfur source. A host of 3-sulfonylated *2H*-indazole derivatives **75** were obtained with moderate to good yields in an undivided cell equipped with a carbon rod anode and a platinum plate cathode at a constant current of 7 mA using LiClO_4_ as the electrolyte (Scheme 16c). A preliminary mechanistic study suggested that this reaction involved the radical-radical cross-coupling between the sulfonyl radical and the *2H*-indazole radical cation, which were generated by anodizing at the anode.

**SCHEME 17:**
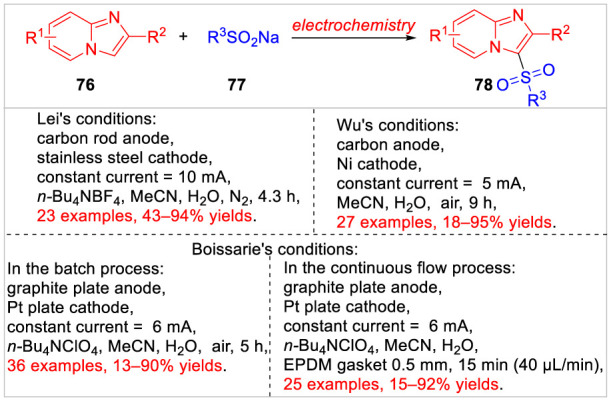
Electrochemical Oxidative C-H Phosphonylation of Thiazole derivatives.

Imidazo [1,2-*a*] pyridines, as a class of nitrogen heterocycles, are widely found in many biologically active compounds and several marketed drugs intermediates (Dymińska, 2015). Therefore, some efficient synthetic methodologies to access polysubstituted imidazo [1,2-*a*] pyridines, especially the electrochemical direct C-H bond functionalization (Wen et al., 2020; Chen et al., 2021), have been reported so far (Scheme 17). In 2021, the Lei group (Zhu et al., 2021) successively reported a metal catalyst-free electrochemical C3-sulfonylation of imidazo [1,2-*a*] pyridines **76** with sodium benzenesulfinates **77**. A series of 3-(arylsulfonyl) imidazo [1,2-*a*] pyridines **78** were efficiently obtained with moderate to high yields in an undivided cell equipped with a carbon rod anode and a stainless steel cathode at a constant current of 10 mA using *n-*Bu_4_NBF_4_ as the electrolyte. Based on controlled experiments, the authors proposed that this reaction involved an arylsulfonyl radical pathway. The same reaction was also realized under electrochemical conditions by Kim, Wu and co-workers (Han et al., 2021). The sulfonylation reaction was conducted in an undivided cell equipped with a carbon anode and a Ni cathode at a constant current of 5 mA in the absence of the electrolyte. Besides, the sodium alkyl sulfinate was suitable for the electrochemical method to produce the desired product in 81% yield. Subsequently, this electrochemical sulfonylation of imidazoheterocycles was developed by Boissarie and co-workers (Leclercq et al., 2021) through batch and continuous flow conditions. The selective C-H bond functionalization tolerated a wide range of functional groups. For example, various sodium sulfinates as well as imidazo [1,2-*a*]-pyridines, -pyrimidine, -quinolines, and -isoquinolines, imidazo [1,2-*b*]pyridazine, imidazo [2,1-*b*] thiazoles and benzo [*d*] imidazo [1,2-*b*] thiazoles could be smoothly transformed into the desired products with moderate to excellent yields. It was worth mentioning that comparing batch conditions, the continuous flow conditions significantly decreased the reaction time while increased the yields.

**SCHEME 18:**
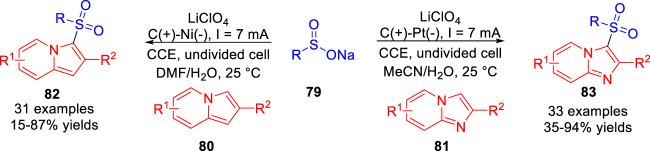
Electrochemical Oxidative C-H Thiolation of Electron-rich Arenes.

In 2021, Oh and co-workers (Kim et al., 2021) also developed an electrochemical oxidative radical C-H sulfonylation of imidazopyridines **81** and indolizines **80** under mild redox reagent-free and metal catalyst-free reaction conditions. A series of sulfonylated *N*-heteroarenes were achieved in moderate to good yields in an undivided cell equipped with a carbon rod anode and a platinum plate cathode/a nickel plate cathode at a constant current of 7 mA using LiClO_4_ as the electrolyte (Scheme 18). Based on controlled experiments, the authors proposed that the oxidation potential-guided reaction may undergo a process of radical-radical cross-coupling between the sulfonyl radical and the imidazopyridine radical cation/indolizine radical cation.

**SCHEME 19:**
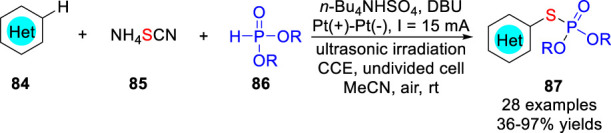
TEMPO-Catalyzed Electrochemical C-H Thiolation of Thioamides.

Also in 2021, the Feng, Xu and co-workers (Meng et al., 2021b) developed a three-component electrochemical oxidative cross-coupling strategy to achieve regioselective C-H phosphorothiolation of (hetero) arenes **84** under ultrasonic irradiation using thiocyanate **85** as the S source. The C-H phosphorothiolation reaction was significantly accelerated by the synergistic cooperation of electrooxidation and ultrasonication. Different kinds of *S*-(hetero) aryl phosphorothioates **87** were synthesized efficiently in an undivided cell equipped with two platinum electrodes at a constant current of 15 mA using *n*-Bu_4_NHSO_4_ as the electrolyte and 1,8-diazabicyclo [5.4.0] undec-7-ene (DBU) as the base under ultrasonic irradiation (Scheme 19). However, substrates having higher oxidation potentials, such as *N*-Boc indole, *N*-Ts pyrrole, and imidazole, were not compatible with this reaction system. On the basis of the control experiments and CV studies, the thiocyanated imidazo [1,2-*a*] pyridine was a key intermediate under electrochemical conditions, which was then attacked by the nucleophilic phosphite to form the desired product.

**SCHEME 20:**
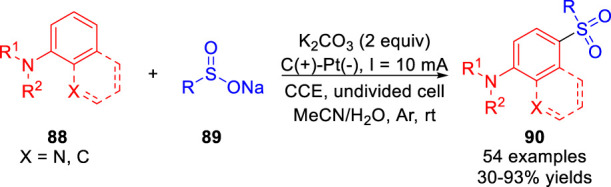
Electrochemical Oxidative C-H Sulfonylation of Arenes/heteroarenes.

Recently, an efficient electrochemical oxidative *para*-selective sulfonylation of *N*-arylamides/amine **88** with sodium sulfinates **89** was realized by Zhang and co-workers (Yu et al., 2022) in the absence of any additional catalyst and oxidant. And various sulfonylated products **90** were efficiently obtained with moderate to high yields in an undivided cell equipped with a graphite rod anode and a platinum plate cathode at a constant current of 10 mA in the absence of the electrolyte (Scheme 20). Moreover, this reaction tolerated a wide range of functional groups, such as aliphatic or aromatic amides, primary amine, secondary and tertiary amines. The authors proposed that the reaction underwent a process of radical-radical cross-coupling between the sulfonyl radical and the C-centered radical resonated from the N-centered radical. It is obvious that the *para*-selective products were obtained due to the *para*-directing amino group.

### C-halide bond formation

Halogenated organic compounds, especially halogenated heterocycles, are significant scaffolds frequently found in bioactive natural products and pharmaceutical industries (Wilcken et al., 2013), and also serve as versatile building blocks for the construction of various complex molecules in coupling reactions (Ruiz-Castillo and Buchwald, 2016). Therefore, halogenation reactions of nitrogen-containing heterocycles have attracted the interest of many medicinal chemists. Here, we mainly introduce the electrochemical catalyst- and oxidant-free approach for direct C-H halogenation of nitrogen heterocycles.

**SCHEME 21:**
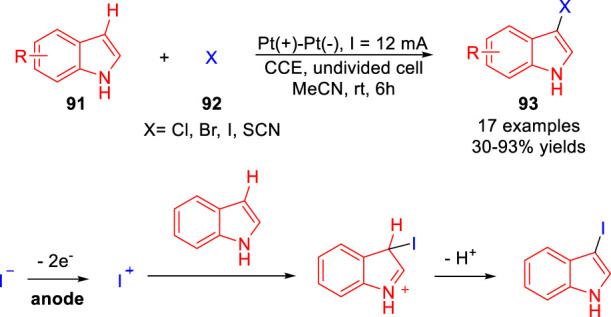
Electrochemical Oxidative C-H Sulfonylation of 2H-indazoles.

In 2018, Jiang and co-workers (Sun et al., 2018) reported an efficient and regioselective electrochemical 3C-H halogenation of indole derivatives **91** under catalyst- and oxidant-free conditions. A series of 3-iodoindole derivatives **93** were efficiently obtained with moderate to high yields in an undivided cell equipped with two platinum electrodes at a constant current of 12 mA using inexpensive KI as the sole reagent. In this transition, potassium iodide plays a dual role, both as a source of iodine and as a supplementary electrolyte. Besides, 3-bromo, 3-chloro-, and 3-thiocyanoindole could also be successfully obtained by introducing the corresponding (pseudo)halogen source under mild conditions (Scheme 21). As expected, the fluorination failed in this transition owing to the extremely high oxidation potential of the fluoride ion. According to control experiments, the authors proposed that the reaction underwent a process of iodide cations.

**SCHEME 22:**
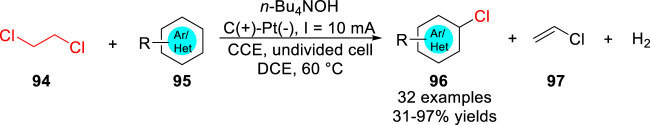
Electrochemical Oxidative C-H Sulfonylation of Imidazoheterocycles.

A year later, a bifunctional electrochemical strategy for the preparation of (hetero) aryl chlorides **96** and vinyl chloride **97** with 1,2-dichloroethane **94** was realized by Jiao et al. under mild conditions (Liang et al., 2019). Various (hetero)aryl chlorides **96** were obtained with moderate to good yields in an undivided cell equipped with a graphite anode and a platinum plate cathode at a constant current of 10 mA using *n*-Bu_4_NOH as the electrolyte (Scheme 22). For example, pyrazole, imidazole, indoles, and some pyridine and quinolone anilides all were compatible with the reaction system to transform the corresponding products. This chlorination reaction involved the dehydrochlorination of DCE at the cathode simultaneously with anodic oxidative aromatic chlorination using the released HCl as the chloride source. The scope of the substrates showed that the substituent positioning effect was in effect. Chlorine was substituted at the *para*-position of the *para*-directing groups, such as amino group or methoxy group, and the *meta*-position of the *meta*-directing group, such as chlorine or trifluoromethyl group.

**SCHEME 23:**
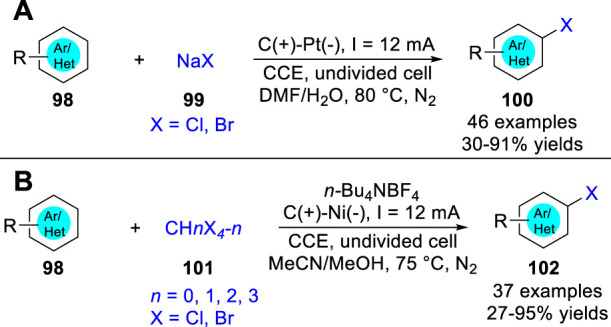
Electrochemical Oxidative C-H Sulfonylation of N-Heteroarenes.

In 2019, Lei and co-workers (Yuan et al., 2019b) reported an electrochemical oxidative clean halogenation using the NaX **99** as a halogen source under metal catalyst- and exogenous oxidant-free reaction conditions. Various (hetero) arenes **98** all were suitable for this reaction system to convert into the corresponding halogenation products **100** by introducing the corresponding halogen source (Scheme 23a). Besides, some alkenes, alkynes, and aliphatic hydrocarbons were also suitable, giving the desired C-Br bond forming products in moderate to high yields under mild electrochemical conditions. Based on control experiments, the reaction mechanism for C-H halogention of heteroarenes was thought to involve chlorine radical or molecular Br_2_, which was directly oxidized at the anode, respectively. Also in 2019, this group (Zhou et al., 2019) also realized the activation of CBr_4_, CHBr_3_, CH_2_Br_2_, CCl_3_Br and CCl_4_ to form C-X bond under electrochemical conditions. And a series of halogenated products **102** were obtained with moderate to excellent yields in an undivided cell equipped with a carbon rod anode and nickel plate cathode at a constant current of 12 mA using *n*-Bu_4_NBF_4_ as the electrolyte by introducing the corresponding halogen source (Scheme 23b). The substrate of s.

**SCHEME 24:**
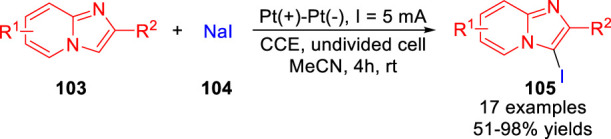
Electrochemical Oxidative C-H Iodination of Imidazo[1,2-a]pyridines.

In 2020, Kim and co-workers (Park et al., 2020) described an electrochemical oxidative iodination of imidazo [1,2-*a*] pyridines **103** using NaI **104** as iodine source and supporting electrolyte under metal catalyst-free and exogenous chemical oxidant-free reaction conditions. A series of 3-iodoimidazo [1,2-*a*] pyridines derivatives **105** were obtained with moderate to excellent yields in an undivided cell equipped with two platinum electrodes at a constant current of 5 mA in acetonitrile at room temperature (Scheme 24). Besides, the bromination of 2-phenylimidazo [1,2-*a*] pyridine with NaBr as halogen source has been successfully realized to form the corresponding C-3 brominated product in 94% yield under mild electrochemical conditions.

## Conclusion

In this mini-review, the metal catalyst-free and exogenous chemical oxidant-free electrochemical C-H bond functionalization of nitrogen heterocycles was briefly described, especially when it could come to functionalize nitrogen heterocycles at different positions. A variety of reactions including formations of C-C, C-N, C-O, C-P, C-S, C-halide bonds have been successfully achieved in an environmentally friendly way from unfunctionalized nitrogen heterocycles by using mild electrochemical conditions. Several advantages of the electrochemical C-H functionalization strategies have been demonstrated. First, some typical oxidative or reductive transformations could be achieved since the reaction reactivity could be tuned by changing the electric current. Second, the product regioselective could also be improved by reducing the electric current that retarded the generation rate of the anodic oxidation.

It is worth mentioning that the use of electrons avoids overoxidation or overreduction caused by stoichiometric chemical reagents in these reactions. Optimization of solvents, electrolytes and conducting experiments under nitrogen protection are also useful methods which could stabilizing free radicals to avoid the overoxidation or overreduction problem. Moreover, through cyclic voltammetry experiments, the appropriate current intensity was selected to solve the problem that the redox potential of products and starting materials is very close.

Looking forward, there are still some challenges to electrochemical C-H bond functionalization of nitrogen heterocycles, including as below: 1) transformations involving heterocycles with 3 or more nitrogen atoms are less developed; 2) how to achieve the combinations of external energy such as photo-irradiation or ultrasonic irradiation with the electrochemical C-H functionalization, which will be more efficient in organic synthesis; 3) the asymmetric electrochemical C-H functionalization of nitrogen heterocycles is rarely developed. With regard to these challenges, we hope that the explanation of metal catalyst-free electrochemical C-H bond functionalization of nitrogen heterocycles in this mini-review will provide useful guidance for further development of other electrochemical C-H bond functionalization.

## References

[B1] Budnikova Y. H. (2021). Electrochemical insight into mechanisms and metallocyclic intermediates of C−H functionalization. Chem. Rec. 21, 2148–2163. 10.1002/tcr.202100009 33629800

[B2] Chen J. F. Yang H. J. Zhang M. F. Chen H. Liu J. Yin K. (2021). Electrochemical-induced regioselective C-3 thiocyanation of imidazoheterocycles with hydrogen evolution. Tetrahedron Lett. 65, 152755–152759. 10.1016/j.tetlet.2020.152755

[B3] Chupakhin O. N. Shchepochkin A. V. Charushin V. N. (2020). A review on application of electrochemical oxidative methods in the C(*sp* ^2^)-H functionalization of heterocyclic compounds. Adv. Heterocycl. Chem. 131, 1–47. 10.1016/bs.aihch.2019.11.002

[B4] Davies D. L. Macgregor S. A. McMullin C. L. (2017). Computational studies of carboxylate-assisted C–H activation and functionalization at group 8–10 transition metal centers. Chem. Rev. 117, 8649–8709. 10.1021/acs.chemrev.6b00839 28530807

[B5] Demmer C. S. Larsen N. K. Bunch L. (2011). Review on modern advances of chemical methods for the introduction of a phosphonic acid group. Chem. Rev. 111, 7981–8006. 10.1021/cr2002646 22010799

[B6] Deng Y. Lu F. L. You S. Q. Xia T. R. Zheng Y. F. Lu C. F. (2019). External-oxidant-free electrochemical oxidative trifluoromethylation of arenes using CF_3_SO_2_Na as the CF_3_ Source. Chin. J. Chem. 37, 817–820. 10.1002/cjoc.201900168

[B7] Dymińska L. (2015). Imidazopyridines as a source of biological activity and their pharmacological potentials—infrared and Raman spectroscopic evidence of their content in pharmaceuticals and plant materials. Bioorg. Med. Chem. 23, 6087–6099. 10.1016/j.bmc.2015.07.045 26314922

[B8] Fu N, K. Li L. J. Yang Q. Luo S. Z. (2017). Catalytic asymmetric electrochemical oxidative coupling of tertiary amines with simple ketones. Org. Lett. 19, 2122–2125. 10.1021/acs.orglett.7b00746 28394132

[B9] Gao P S P. S. Weng X. J. Wang Z. H. Zheng C. Sun B. Chen Z. H. (2020). Cu^II/^TEMPO-catalyzed enantioselective C(*sp* ^3^)-H alkynylation of tertiary cyclic amines through shono-type oxidation. Angew. Chem. Int. Ed. 59, 15254–15259. 10.1002/anie.202005099 32394631

[B10] Gao W. J. Li W. C. Zeng C. C. Tian H. Y. Hu L. M. Little R. D. (2014). Electrochemically initiated oxidative amination of benzoxazoles using tetraalkylammonium halides as redox catalysts. J. Org. Chem. 79, 9613–9618. 10.1021/jo501736w 25255384

[B11] Gao Y Y Y. Y. Wu Z. G. Yu L. Wang Y. Pan Y. (2020). Alkyl carbazates for electrochemical deoxygenative functionalization of heteroarenes. Angew. Chem. Int. Ed. 59, 10859–10863. 10.1002/anie.202001571 32227611

[B12] Goel R. Luxami V. Paul K. (2016). A review on imidazo[1, 2-*a*]pyridines: Promising drug candidate for antitumor therapy. Curr. Top. Med. Chem. 16, 3590–3616. 10.2174/1568026616666160414122644 27086790

[B13] Han L. L. Huang M. M. Li Y. B. Zhang J. Y. Zhu Y. Kim J. K. (2021). An electrolyte- and catalyst-free electrooxidative sulfonylation of imidazo[1, 2-*a*]pyridines. Org. Chem. Front. 8, 3110–3117. 10.1039/D1QO00038A

[B14] Jiang Y. Y. Dou G. Y. Zhang L. S. Xu K. Little R. D. Zeng C. C. (2019). Electrochemical cross-coupling of C(*sp* ^2^)-H with aryldiazonium salts via a paired electrolysis: An alternative to visible light photoredox-based approach. Adv. Synth. Catal. 361, 5170–5175. 10.1002/adsc.201901011

[B15] Jiang Y. Y. Xu K. Zeng C. C. (2018). Use of electrochemistry in the synthesis of heterocyclic structures. Chem. Rev. 118, 4485–4540. 10.1021/acs.chemrev.7b00271 29039924

[B16] Kakiuchi F. Kochi T. (2020). New strategy for catalytic oxidative C–H functionalization: Efficient combination of transition-metal catalyst and electrochemical oxidation. Chem. Lett. 49, 1256–1269. 10.1246/cl.200475

[B17] Kim W. Kim H. Y. Oh K. (2020). Electrochemical radical-radical cross-coupling approach between sodium sulfinates and *2H*-Indazoles to 3-sulfonylated *2H*-Indazoles. Org. Lett. 22, 6319–6323. 10.1021/acs.orglett.0c02144 32806182

[B18] Kim W. Kim H. Y. Oh K. (2021). Oxidation potential-guided electrochemical radical-radical cross-coupling approaches to 3-sulfonylated imidazopyridines and indolizines. J. Org. Chem. 86, 15973–15991. 10.1021/acs.joc.1c00873 34185997

[B19] Leclercq E. Boddaert M. Beaucamp M. Penhoat M. Boissarie L. C. (2021). Electrochemical sulfonylation of imidazoheterocycles under batch and continuous flow conditions. Org. Biomol. Chem. 19, 9379–9385. 10.1039/D1OB01822A 34673877

[B20] Li J. J. Zhang S. Xu K. (2021). Recent advances towards electrochemical transformations of α-keto acids. Chin. Chem. Lett. 32, 2729–2735. 10.1016/j.cclet.2021.03.027

[B21] Li K. J. Jiang Y. Y. Xu K. Zeng C. C. Sun B. G. (2019). Electrochemically dehydrogenative C-H/P-H cross-coupling: Effective synthesis of phosphonated quinoxalin-2(*1H*)-ones and xanthenes. Green Chem. 21, 4412–4421. 10.1039/c9gc01474h

[B22] Liang Y. J. Lin F. G. R. Adeli Y. Jin R. Jiao N. (2019). Efficient electrocatalysis for the preparation of (hetero)aryl chlorides and vinyl chloride with 1, 2-dichloroethane. Angew. Chem. Int. Ed. 58, 4566–4570. 10.1002/anie.201814570 30664331

[B23] Liang Y. W. Niu L. B. Liang X. A. Wang S. C. Wang P. J. Lei A. W. (2022). Electrooxidation-induced C(*sp* ^3^)-H/C(*sp* ^2^)-H radical-radical cross-coupling between xanthanes and electron-rich arenes. Chin. J. Chem. 40, 1422–1428. 10.1002/cjoc.202200020

[B24] Luo M. J. Ouyang X. H. Zhu Y. P. Li Y. Li J. H. (2021). Metal-free electrochemical [3 + 2] heteroannulation of anilines with pyridines enabled by dual C-H radical aminations. Green Chem. 23, 9024–9029. 10.1039/D1GC02922C

[B25] Meng Z. Y. Feng C. T. Xu K. (2021a). Recent advances in the electrochemical formation of carbon-nitrogen bonds. Chin. J. Org. Chem. 41, 2535–2570. 10.6023/cjoc202012013

[B26] Meng Z. Y. Feng C. T. Zhang L. Yang Q. Chen D. X. Xu K. (2021b). Regioselective C-H phosphorothiolation of (hetero)arenes enabled by the synergy of electrooxidation and ultrasonic irradiation. Org. Lett. 23, 4214–4218. 10.1021/acs.orglett.1c01161 33983749

[B27] Ohno S. Miyoshi M. Murai K. Arisawa M. (2021). A review on non-directed β- or γ-C(*sp* ^3^)-H functionalization of saturated nitrogen-containing heterocycles. Synthesis 53, 2947–2960. 10.1055/a-1483-4575

[B28] Park J. W. Kim Y. H. Kim D. Y. (2020). Electrochemical oxidative iodination of imidazo[1, 2-*a*]pyridines using NaI as iodine source. Synth. Commun. 50, 710–718. 10.1080/00397911.2020.1717539

[B29] Pastor M. Vayer M. Weinstabl H. Maulide N. (2022). Electrochemical umpolung C-H functionalization of oxindoles. J. Org. Chem. 87, 606–612. 10.1021/acs.joc.1c02616 34962127 PMC8749966

[B30] Peng X. C. Zhao J. H. Ma G. J. Wu Y. Hu S. Y. Ruan Z. X. (2021). Electro-oxidative C-H amination of heteroarenes with aniline derivatives via radical-radical cross coupling. Green Chem. 23, 8853–8858. 10.1039/D1GC02821A

[B31] Qian X. Y. Li S. Q. Song J. S. Xu H. C. (2017). TEMPO-Catalyzed electrochemical C-H thiolation: Synthesis of benzothiazoles and thiazolopyridines from thioamides. ACS Catal. 7, 2730–2734. 10.1021/acscatal.7b00426

[B32] Qiu Y. A. Struwe J. L. Meyer T. H. Oliveira J. C. A. Ackermann L. (2018). Catalyst- and reagent-free electrochemical azole C-H amination. Chem. Eur. J. 24, 12784–12789. 10.1002/chem.201802832 29901828

[B33] Ruiz-Castillo P. Buchwald S. L. (2016). Applications of palladium-catalyzed C–N cross-coupling reactions. Chem. Rev. 116, 12564–12649. 10.1021/acs.chemrev.6b00512 27689804 PMC5070552

[B34] Sharma A. Vacchani D. Van der Eycken E. (2013). Developments in direct C–H arylation of (Hetero)Arenes under microwave irradiation. Chem. Eur. J. 19, 1158–1168. 10.1002/chem.201201868 23293098

[B35] Sun L. H. Zhang X. Li Z. L. Ma J. M. Zeng Z. Jiang H. (2018). A versatile C-H halogenation strategy for indole derivatives under electrochemical catalyst- and oxidant-free conditions. Eur. J. Org. Chem. 4949–4952. 10.1002/ejoc.201800267

[B36] Tan Z, M. He X. R. Xu K. Zeng C. C. (2022). Electrophotocatalytic C-H functionalization of *N*-Heteroarenes with unactivated alkanes under external oxidant-free conditions. ChemSusChem 15, e202102360. 10.1002/cssc.202102360 34967138

[B37] Vitaku E. Smith D. T. Njardarson J. T. (2014). Analysis of the structural diversity, substitution patterns, and frequency of nitrogen heterocycles among U.S. FDA approved pharmaceuticals. J. Med. Chem. 57, 10257–10274. 10.1021/jm501100b 25255204

[B38] Wang J. H. Li X. B. Li J. Lei T. Wu H. L. Nan X. L. (2019). Photoelectrochemical cell for P-H/C-H cross-coupling with hydrogen evolution. Chem. Commun. 55, 10376–10379. 10.1039/c9cc05375a 31386711

[B39] Wang P. Tang S. Huang P. F. Lei A. W. (2017). Electrocatalytic oxidant-Free dehydrogenative C-H/S-H cross-coupling. Angew. Chem. Int. Ed. 56, 3009–3013. 10.1002/anie.201700012 28177563

[B40] Wen J. W. Niu C. Yan K. L. Cheng X. D. Gong R. K. Li M. Q. (2020). Electrochemical-induced regioselective C-3 thiomethylation of imidazopyridines via a three-component cross-coupling strategy. Green Chem. 22, 1129–1133. 10.1039/c9gc04068d

[B41] Wilcken R. Zimmermann M. O. Lange A. Joerger A. C. Boeckler F. M. (2013). Principles and applications of halogen bonding in medicinal chemistry and chemical biology. J. Med. Chem. 56, 1363–1388. 10.1021/jm3012068 23145854

[B42] Xie W. X. Liu N. Gong B. W. Ning S. L. Che X. Cui L. L. (2019). Electrochemical cross-dehydrogenative coupling of *N*-aryl-tetrahydroisoquinolines with phosphites and indole. Eur. J. Org. Chem. 14, 2498–2501. 10.1002/ejoc.201801883

[B43] Yu Y. L. Fang Y. Tang R. Xu D. P. Dai S. S. Zhang W. (2022). Electrochemical oxidative sulfonylation of *N*-arylamides/amine with sodium sulfinates. Asian J. Org. Chem. 11, e202100805. 10.1002/ajoc.202100805

[B44] Yuan Y. Qiao J. Cao Y. M. Tang J. M. Wang M. Q. Ke G. J. (2019a). Exogenous-oxidant-free electrochemical oxidative C-H phosphonylation with hydrogen evolution. Chem. Commun. 55, 4230–4233. 10.1039/c9cc00975b 30899925

[B45] Yuan Y. Yao A. J. Zheng Y. F. Gao M. Zhou Z. L. Qiao J. (2019b). Electrochemical oxidative clean halogenation using HX/NaX with hydrogen evolution. iScience 12, 293–303. 10.1016/j.isci.2019.01.017 30735897 PMC6365813

[B46] Yuan Y. Yu Y. Qiao J. Liu P. Yu B. Y. Zhang W. K. (2018). Exogenous-oxidant-free electrochemical oxidative C-H sulfonylation of arenes/heteroarenes with hydrogen evolution. Chem. Commun. 54, 11471–11474. 10.1039/C8CC06451B 30255875

[B47] Zhang H. Lei A. W. (2019)., 51. Synthesis, 83–96. 10.1055/s-0037-1610380 Electrochemical/photochemical aminations based on oxidative cross-coupling between C–H and N–H Synthesis

[B48] Zhang Y. Lin Z. P. Ackermann L. (2021). Electrochemical C-H amidation of heteroarenes with *N*-alkyl sulfonamides in aqueous medium. Chem. Eur. J. 27, 242–246. 10.1002/chem.202004229 33085807 PMC7898600

[B49] Zhao H. B. Hou Z. W. Liu Z. J. Zhou Z.-F. Song J. Xu H. C. (2017). Amidinyl radical formation through anodic N-H bond cleavage and its application in aromatic C-H bond functionalization. Angew. Chem. Int. Ed. 56, 587–590. 10.1002/anie.201610715 27936308

[B50] Zhao H. B. Xu P. Song J. S. Xu H. C. (2018). Cathode material determines product selectivity for electrochemical C-H functionalization of biaryl ketoximes. Angew. Chem. Int. Ed. 57, 15153–15156. 10.1002/anie.201809679 30225909

[B51] Zhou N. F. Yu J. C. Hou L. Y. Wu X. Ruan Z. X. Feng P. J. (2022). Electro-oxidative coupling of azoles with 2- and 3-haloindoles/thiophenes providing access to 2/3-halo(azol-1-Yl)indoles/thiophenes. Adv. Synth. Catal. 364, 35–40. 10.1002/adsc.202100958

[B52] Zhou Z. L. Yuan Y. Cao Y. M. Qiao J. Yao A. J. Zhao J. (2019). Synergy of anodic oxidation and cathodic reduction leads to electrochemical C-H halogenation. Chin. J. Chem. 37, 611–615. 10.1002/cjoc.201900091

[B53] Zhu J. Y. Chen Z. Y. He M. Wang D. X. Li L. S. Qi J. C. (2021). Metal-free electrochemical C3-sulfonylation of imidazo[1, 2-*a*]pyridines. Org. Chem. Front. 8, 3815–3819. 10.1039/D1QO00348H

[B54] Zhu P. W. Yang Y. T. Li Y. Zhu J. Wu L. (2022). Electrochemical oxidative C-H phosphonylation of thiazole derivatives in ambient conditions. Mol. Catal. 517, 112022–112026. 10.1016/j.mcat.2021.112022

